# Reorienting the Debate on Biological Individuality: Politics and Practices: Review of Alison K. McConwell. *Biological Individuality. Elements in the Philosophy of Biology*. Cambridge: Cambridge University Press. 93pp. DOI:https://doi.org/10.1017/9781108942775; ISBN: 9781009387422

**DOI:** 10.1007/s10441-024-09479-9

**Published:** 2024-02-28

**Authors:** Rose Trappes

**Affiliations:** University of Exeter, Exeter, UK

**Keywords:** Biological individuality, Organism, practice-based philosophy of science, Political philosophy of science, Pluralism, Feminist philosophy of science

## Abstract

Biological individuality is without a doubt a key concept in philosophy of biology. Questions around the individuality of organisms, species, and biological systems can be traced throughout the philosophy of biology since the discipline’s inception, not to mention the sustained attention they have received in biology and philosophy more broadly. It’s high time the topic got its own Cambridge Element. McConwell’s *Biological Individuality* falls short of an authoritative overview of the debate on biological individuality. However, it sends a welcome message to new and seasoned scholars to reorient the debate towards practically and politically relevant themes.

## A Call to Change Course

Over the last two decades, a relatively well-defined debate on biological individuality has arisen within philosophy of biology. This debate is broadly concerned with questions related to what makes some biological entity an individual (Kaiser and Trappes 2021). Philosophers discuss concepts and definitions of individuality in relation to evolutionary biology but also disciplines like immunology, developmental biology, microbiology, and ecology. They develop theories regarding the evolution of new levels of individuality, such as multicellularity or complex forms of cooperation. And they debate whether there are many best ways of carving up the living world into individuals, and how this pluralism might map onto disciplinary divides or domains of life.

Alison McConwell’s *Biological Individuality* (2023) is best read as an impulse to this debate, a call to reorient philosophical investigations away from quibbling over problem cases and definitions and towards considering the epistemic, ethical and political implications of concepts of biological individuality. In doing so, McConwell builds explicitly on the practice turn that the debate on biological individuality has undergone in the last decade (45 − 7). The practice turn, McConwell rightly insists, implies considering the usefulness of concepts of individuality for biologists in many different disciplines, the role of ideology and political imaginaries in conceptualising individuals in the living world, and the ethical implications of assigning entities the status of individuality. Philosophers working on biological individuality, in other words, need to get their hands dirty; philosophy of biological individuality needs to get practical and political.

In sending this message, McConwell hopes to advise students and junior scholars, as well as more advanced scholars already invested in the topic. She writes candidly about her motivation: “As a graduate student, I found the topic very complicated and difficult. The sections of the Element are written in a way that draws from what I wish I would have known and where I hope to see work go in the future.” (3) McConwell’s engaging style—unfortunately somewhat hampered by poor copy editing and by several incongruous engagements with an anonymous reviewer—helps lend the text a pedagogical feel, as does the use of figures and tables. Noteworthy too are the pointers McConwell gives throughout regarding directions for future research (e.g., 25, 42, 55); these are very valuable in a thoroughly explored area like biological individuality. The advice to engage with practising biologists using qualitative empirical methods (54) is also on trend, though unfortunately not coupled with references for methodological guidance (see, e.g., Wagenknecht et al. 2015; Nersessian and MacLeod 2022; Hangel and ChoGlueck 2023).

*Biological Individuality* works as a platform for guiding the debate away from a “cottage industry” (51) and towards more productive terrain. Its major failing is in its treatment of the literature. There is of course no way a slim Elements volume could cover all important aspects of such a massive and multifaceted debate as the one on biological individuality. I myself have received push-back from players in the debate for supposed failures in treating the literature. I certainly don’t want to pay that on. But the Elements series is explicitly intended to provide authoritative introductions characterised by “balanced, comprehensive coverage of multiple perspectives” (Cambridge Elements in Philosophy of Biology). The treatment in *Biological Individuality* of both historical and contemporary literature on individuality is neither comprehensive nor balanced—as McConwell herself explicitly acknowledges (1). Readers looking for a systematic introduction to the topic (for teaching, say, or for a quick entry into the field) should look elsewhere (e.g., Pradeu 2016; Lidgard and Nyhart 2017a; Wilson and Barker 2019).

## Politics

*Biological Individuality* is valuable for the prominence it grants to ethical and political considerations. McConwell takes care to bring these issues in repeatedly throughout the text, rather than being relegated to a separate section or an afterthought. For instance, readers get not only an excellent overview of David Hull’s seminal work on the individuality of species, but also insights into the political implications of treating species as individuals. As McConwell writes, “Throughout history, many people were dehumanized as deviants from humanity. In response, Hull’s view implies one is human insofar as they are part of the human lineage, rather than satisfying some necessary (set of) features that all and only humans have.” (15) This is an insightful observation that is easy to neglect in favour of purely theoretical considerations.

Similarly progressive politics can be found throughout the history of thought on biological individuality (Nyhart and Lidgard 2021). At the same time, conceptualisations of biological individuality have been informed by and used to support the ideology and practices of eugenics. McConwell astutely identifies this tension in the political meaning of biological individuality, directing philosophers of biology to bear in mind the “dark side” of biological individuality (78).

It is unfortunate that this important reminder comes out of an overly lengthy and somewhat convoluted presentation of Julian Huxley’s views on biological individuality and his links to eugenics. Greater contextualisation and balance would have helped to avoid the impression that Huxley was the only major figure in early 20th century biology thinking about individuality, and to clarify that many biologists at the time applied their theories to social and political issues. Particularly important here is the existing work on the history of individuality and organism concepts and their relation to theoretical and political movements such as reductionism, holism, vitalism, and mechanicism (e.g., Cheung 2010; Wolfe 2010; Lidgard and Nyhart 2017b; Baedke 2019a), not to mention the large amount of scholarship on the history of eugenics in the life sciences.

Some philosophers of biology might be uncomfortable with the idea that they ought to consider the political implications of their work. Surely arguments for democracy or eugenics based on theories of biological individuality are best left in the past? Yet McConwell argues that current work on individuality is not politically neutral. Philosophers of biology should therefore face up to the political implications of their work.

McConwell cites arguments that current philosophy of biology still tends to operate with a colonial logic, which denies truth gluts or true contradictions; this is evident in the (contested) assumption that something either is or is not an individual (Sinclair 2020). Moreover, McConwell suggests that a colonial objectification of nature is evidenced in work about the individuality of ecological systems (48 − 9). In particular, she points out that a clearly delineated status of individuality, separate from human managers, is often seen as necessary for the recognition and protection of ecological systems. This “separates the manager (i.e., humans) as external entities imposing their will often for use and exploitation of the land and by that action objectifies nature.” (49) There is also a provocative discussion of how the individuation of traits or characters relies on positivist standards; what is left unclear is whether these positivist standards are also colonial, as well as exactly how trait individuation and individuality relate. More generally, greater precision regarding the “pillars of modernism—a complex of enlightenment, colonial, and positivist ideals” (51) would have helped provide a stronger starting point for those philosophers embarking on the project of reassessing the politics of their work.

One important locus for thinking about the ethical and political dimensions of theorising about individuality is feminist philosophy of science, science and technology studies (STS) and biopolitics. These areas are often overlooked in mainstream philosophy of biology, making it all the more valuable that McConwell explicitly recognises them as sites for “considering individuality as the complex juncture of bio-social spaces.” (57) McConwell cites two of Donna Haraway’s most well-known texts, “A Cyborg Manifesto” (Haraway 1991) and *The Companion Species Manifesto* (Haraway 2003), using these texts to point out how individuals actively construct their own boundaries in collaboration with other organisms and technology. A few more indications of relevant works in feminist philosophy of science and STS would have been helpful. For instance, there is a rich literature in feminist theory that addresses bodily boundaries, interdependencies, and transcorporeality (reviewed in, e.g., Alaimo and Hekman 2008; Hird 2009). Biopolitics is also a fruitful resource for thinking about the political dimensions of definitions and practices of reproduction, life, and death—all implicated in the concept of individuality (Esposito 2008; Mills 2018).

Scholarship on individuality outside philosophy of biology and analytic metaphysics is in fact vast; other relevant areas include philosophy and sociology coming from French, German, and Italian traditions (e.g., Simondon 1992; Gayon 1998; Beck 2002; Honneth 2004; Hengehold 2017). Many of these areas of research focus on social and political aspects of individuality, such as how to understand individuality while also recognising interdependence and vulnerability, or how social organisation and economic structures can lead to greater individuality amongst members of society. There is much to explore in these fields that could augment the reorientation that McConwell calls for in the philosophy of biological individuality.

## Practices

Few philosophers seek to defend a single definition of biological individuality for all contexts and purposes. Instead, a consensus has emerged in the debate on biological individuality around pluralism (Pradeu 2016). A common version of this pluralism has it that there are different concepts of individuality for different disciplines in biology, perhaps even forming different kinds of biological individuality: evolutionary individuality and physiological individuality, for instance. Others hold that there are many valid concepts of individuality corresponding to different epistemic practices, and especially to different ways of individuating entities in the living world.

For her part, McConwell distinguishes between organismality and individuality, and then between several types of individuality: evolutionary, immunological, metabolic, ecological, and developmental (33–36). The latter “domain-driven” (32) set of distinctions falls mostly in line with other overviews of biological individuality. One of the difficulties of disciplinary or domain-driven pluralism in scientific concepts is how to make sense of interdisciplinary communication and collaboration. Although this challenge is recognised with respect to other scientific concepts (Haueis 2021), it often receives little attention in discussions of individuality. Refreshingly, McConwell does briefly touch on the complexities introduced by vague, ambiguous, and changing disciplinary boundaries (42–43). This will hopefully provide some impetus for research on the connections between individuality concepts across scientific domains.

In contrast to domain-driven pluralism, there is no current consensus about the distinction between organisms and individuals (Prieto 2023). McConwell associates organismality with historical figures, etymology, and the tradition of organicism (4–8). This association tends—perhaps unintentionally—to relegate this concept to biology’s past, which doesn’t do justice to the recent resurgence of the organism across the life sciences (Nicholson 2014; Baedke 2019b; Fábregas-Tejeda and Martín-Villuendas 2023). In addition, the book’s separate presentation of organismality and individuality risks creating the misleading impression that research into autopoiesis, autonomy, and agency does not belong to the debate on biological individuality. As with interdisciplinary connections, there is room for further analysis of how concepts of individuality and organismality interact in different scientific contexts.

McConwell treats evolutionary individuality in particular detail, covering early discussions about species as individuals, as well as more recent work on units of selection and major transitions in evolution. In the process, she introduces further sorts of pluralism. On the one hand, in addition to domain-driven pluralism there can be conceptual pluralism within domains (37). Examples of the latter include recognising both functional and material concepts of evolutionary individuality, or units of evolution (species) as well as units of selection. On the other hand, McConwell introduces a notion of diachronic pluralism, in which new types of individuality emerge (and disappear) over time, especially through evolution (41).

The evolutionary focus is in line with McConwell’s own research trajectory and reflects broader tendencies in philosophy of biology. It does however result in a picture that is skewed towards theoretical philosophy of biology, with less attention devoted to the often more practice-oriented and socially-relevant discussions of physiological, developmental, ecological, and behavioural individuality (Bueno et al. 2018). For example, McConwell does introduce immunological, ecological, and metabolic individuality, including practically important and normatively charged issues such as cancer, organ transplants, ecological conservation, and personalised medicine. Yet these topics receive a scanty five pages (26–31), in contrast to the detailed and diagram-rich 21 pages devoted to topics in evolutionary individuality (8–25; 39–42).

Looking beyond evolutionary individuality, we find an already robust tradition of research into practically and socially relevant aspects of individuality. For instance, McConwell cites holobiont individuality—the question of whether we are multispecies individuals including our microbiomes—as an example of a socially relevant topic that philosophers of biology should address (30; 57 − 8). Fortunately, many philosophers are already debating holobiont individuality in light of its potential theoretical, practical, and ethical consequences (e.g., Chiu and Eberl 2016; Skillings 2016; Kirby 2017; Şencan 2019; Suárez and Stencel 2020; Formosinho et al. 2022). Similarly, McConwell’s proposal to study individuality in synthetic biology and biotechnology could be connected to the vast body of existing work in bioethics about identity in relation to cloning, genetic modification, and gene editing (e.g., Hauskeller 2004; Ankeny and Bray 2018; Douglas and Devolder 2022). Another politically and ethically relevant topic that McConwell fails to mention is the individuality of pregnant organisms and foetuses; again, this is a topic that has received recent attention, for instance in relation to immunological and metabolic criteria of individuality (Kingma 2020; Meincke 2021; Morgan 2022). The project of reorienting the debate on biological individuality can and should build on such existing positive examples.

One of the live questions for practice-based philosophy of science is how to understand the relationship between scientific practice and concepts or ontology (Feest and Steinle 2012). McConwell distinguishes several different practice-based approaches to biological individuality. For instance, some philosophers analyse scientific practices, especially the practices through which scientists individuate organisms or other biological systems, to identify implicit concepts of individuality at work. Others develop individuality concepts with a view to their practical usefulness, for instance for the purposes of counting units of selection. Less clearly practice-based is the study of puzzle cases from biology—biological systems that do not fit our intuitions about individuality, such as huge clonal meadows of sea grass or lichens with their tight symbiotic associations between fungi and algae. McConwell apparently lumps the study of puzzle cases under the practice turn (47), and later argues that such puzzle-driven discourse should be replaced by greater engagement with practising biologists. On the other hand, given that the biologists we interact with may themselves be puzzling over problem cases, puzzle-driven discourse could be here to stay.

The discussion of practice-based conceptual analysis concentrates on the explanatory and practical uses of concepts, skirting around the matter of metaphysics. Perhaps for this reason, important recent work on individuality in relation to process ontology and personal identity go unmentioned (see, e.g., entries in Nicholson and Dupré 2018; Meincke and Dupré 2021). This area of research includes substantial discussions of how to understand the project of practice-based metaphysics of science (see also Bausman et al. 2023). It also makes clear that the debate about biological individuality needn’t restrict itself to epistemology. Biological individuality can act as a starting point for thinking about some of the big issues in contemporary philosophy of science and metaphysics, including pluralism, pragmatism, and perspectival realism.

## Towards a Political, Practice-Based Philosophy of Biological Individuality

I’ve treated politics and practice mostly separately in this review, but the clear message of *Biological Individuality* is that they must be combined in a thoroughly political and practice-based philosophy of science. McConwell frames this project in terms of the epistemic and non-epistemic value of biological individuality. Although canonical in philosophy of science, the distinction between epistemic and non-epistemic values has long been subject to criticism, revision and complexification (Longino 1996). Rather than separating politics and practice, the social and the scientific, we need a framework that recognises their enmeshment. As McConwell argues, this is sure to reinvigorate the debate on biological individuality and connect it to a much wider network of thought on individuality and the life sciences.

## Data Availability

This publication does not make use of any data.
